# Prevalence and clinical correlates of substance use amongst acute psychiatric inpatients in Gauteng, South Africa

**DOI:** 10.4102/sajpsychiatry.v26i0.1526

**Published:** 2020-09-25

**Authors:** Ani Anic, Lesley J. Robertson

**Affiliations:** 1 Department of Psychiatry, School of Clinical Medicine, University of the Witwatersrand, Johannesburg, South Africa; 2 Charlotte Maxeke Johannesburg Academic Hospital, Johannesburg, South Africa; 3 Sedibeng District Health Services, Vanderbijlpark, South Africa

**Keywords:** substance use, AUDIT, DUDIT, Helen Joseph Hospital, mental disorders

## Abstract

**Background:**

Mental disorders and substance use disorders (SUD) commonly occur together, impacting healthcare outcomes. The diagnosis of substance use is often inadequate when comorbidity is present. It is vital to understand the prevalence of substance use amongst psychiatric patients to inform both clinical practice and service development in South Africa.

**Aim:**

To ascertain the prevalence and clinical correlates of SUD amongst acute psychiatric inpatients.

**Setting:**

The setting for this study was Helen Joseph Hospital acute psychiatric ward.

**Methods:**

A cross-sectional study was conducted whereby consecutively admitted patients were invited to participate in a structured clinical interview utilising the alcohol use disorders identification test (AUDIT) and drug use disorders identification test (DUDIT) questionnaires. Statistical comparisons were made between those with and without SUD.

**Results:**

Of 150 participants, 100 (67%) were identified with a SUD. Those with SUD were younger (*p* = 0.0010), more often male (*p* = 0.012), less likely to have a disability grant (*p* = 0.015) and more likely to be brought to hospital by police, ambulance or self than by a family member (*p* = 0.025). Almost half of people with bipolar disorder (47.3%) and schizophrenia (41.4%) had comorbid SUD. Twenty-three (15%) participants identified with SUD on questionnaire had been missed clinically. Only two participants were referred for inpatient substance rehabilitation on discharge.

**Conclusion:**

Substance use disorders are highly prevalent amongst psychiatric inpatients. The AUDIT and DUDIT are potentially useful screening tools in routine clinical practice. Greater collaboration between psychiatric and substance rehabilitation services is recommended.

## Introduction

Mental illness and substance use disorders (SUD) are estimated to affect 16% of the world’s population^1^ and account for almost 19% of the global burden of disease because of years lived with disability. In addition, they confer an indirect impact on mortality, which suggests a greater burden than what prevalence rates indicate.^1^ Recognising the need to address these conditions, the United Nations has included them in the Sustainable Development^2^ Goals, with health target 3.4: ‘by 2030, reduce by one-third premature mortality from non-communicable diseases through prevention and treatment and promote mental health and well-being’; and health target 3.5: ‘strengthen the prevention and treatment of substance use, including narcotic drug abuse and harmful use of alcohol’.^2^

There is a well-established relationship between mental disorders and SUD, including high levels of comorbidity recorded between the two conditions,^3,4,5,6,7^ increased psychiatric symptom severity,^4^ poor care outcomes, more impaired functioning,^7^ poor medication adherence and treatment dropout.^8^ Increased hospitalisation rates, with shorter duration of stay, have been reported in patients with comorbid SUD and bipolar disorder^4^ or schizophrenia.^9^ Having a SUD increases the risk of violent behaviour in community and acute inpatient settings^10,11,12,13^ and, in South Africa, it is associated with a longer duration of admission amongst male state patients.^14^ Finally, suicidal behaviour, completed suicide and all-cause mortality are all increased in the presence of a comorbid SUD.^15,16,17,18,19,20^

Population surveys^3,6^ have revealed high prevalence rates of mental illness among substance users, ranging from double that of the general population for anxiety disorders to five times that of the general population for bipolar disorder. When looking at SUD amongst persons with mental illness, a recent systematic review of SUD amongst people with bipolar disorder found prevalence figures (including lifetime and current rates) of 42% for alcohol use, 20% for cannabis use and 17% for other recreational drugs.^4^ Schizophrenia is similarly associated with a high overall prevalence of SUD (41.7% for any SUD, 27.5% for recreational drugs, 26.2% for cannabis and 24.3% for alcohol), which has largely remained stable over time.^5^

There is a need to establish prevalence rates of substance use amongst psychiatric patients in South Africa to inform both clinical practice and service development. However, Morojele, Saban and Seedat^7^ reported that the diagnosis of SUD amongst people with mental illness is often inadequate because of the heterogeneity in clinical presentation and symptom severity. They further commented that the use of inadequate diagnostic approaches contributes to low levels of detection and recommended the need for more routine application of standardised screening tools.

Using their own data collection tool in a prospective survey amongst acute admissions at a psychiatric hospital, Weich and Pienaar^21^ found a prevalence rate of 51% for comorbid current SUD. Two recent cross-sectional studies in Africa using the World Health Organization’s (WHO) Alcohol, Smoking, and Substance Involvement Screening Test (ASSIST) amongst people with severe mental illness reported the following prevalence rates: Davis et al.^22^ in a regional general hospital in South Africa found lifetime substance use in 90% of patients, whereas Sowumni et al.^23^ reported a lifetime prevalence of 47% and a current prevalence of 17% amongst outpatients at a neuropsychiatric specialised hospital in Nigeria.

The primary aim of this study was to determine the prevalence of SUD using standardised screening instruments, and to describe their clinical correlates, amongst patients admitted to an acute psychiatric ward in a general hospital. A secondary objective was to compare the prevalence rates of SUD according to the clinical records with prevalence rates when using screening questionnaires. In terms of clinical presentation, we hypothesised that those with SUD would show higher rates of psychosis and aggressive symptoms.

## Methods

A cross-sectional study was conducted amongst patients admitted to the acute psychiatric unit at Helen Joseph Hospital, a tertiary academic general hospital in Johannesburg, Gauteng province, South Africa. The unit is a 30-bed, mixed male and female, acute adult assessment ward accepting referrals from surrounding clinics as well as the casualty. The city areas served by the hospital are well known for a high level of crime and drug-related problems.^24^

The inclusion criteria required the participants to be 18 years or older, conversant in English or Afrikaans, and have capacity to consent. All patients admitted to the unit between 01 February and 31 May 2016 were approached by the principal investigator (A.A.) as soon as they were clinically stable enough to provide informed consent.

### Study tools

Following written informed consent, the patients were interviewed and the socio-demographic and clinical information, in accordance with a questionnaire developed by the researcher (A.A.), was obtained. The socio-demographic details obtained included gender, race, relationship status, highest level of education, employment status, whether receiving a disability grant, religion and handedness.

This was then followed by the administration of both the alcohol use disorders identification test (AUDIT) and drug use disorders identification test (DUDIT) by the principal investigator. The hospital’s clinical records were used to establish the date of admission and discharge, admission status in accordance with the *Mental Health Care Act*, who had brought them to hospital, presenting symptoms, the discharge diagnosis (including medical, substance-related and psychiatric diagnoses), treatment prescribed, number of psychiatric admissions and disposition following the current admission.

The AUDIT and DUDIT questionnaires have both been confirmed as valid and reliable tools amongst acute psychiatric inpatients internationally^25^ and have also been validated in South Africa.^26^ The questions are based on Diagnostic and Statistical Manual of Mental Disorders, Fourth edition (DSM-IV) and International Classification of Diseases, tenth revision (ICD-10) criteria for substance abuse and dependence. The AUDIT was developed by the WHO as a screening tool to identify people with hazardous, harmful or dependent alcohol use and has a sensitivity of 83% and specificity of 90% when tested against DSM diagnostic criteria.^27^ As recommended by the AUDIT manual, cut-off scores of 8 for men and 6 for women were used to identify hazardous or harmful alcohol use and a score of 20 or more for both sexes as an indication of alcohol dependence.^27^ The DUDIT was developed as a parallel instrument to the AUDIT for identification of drug use other than alcohol, with a sensitivity of 90% and specificity of 78%.^28^ As recommended by the DUDIT manual, cut-off scores of 6 for men and 2 for women were used to identify hazardous or harmful drug use, and a score of 25 or more for both sexes as an indication of drug dependence.^28^ The tools have been validated in English, with the investigator translating to Afrikaans for those needed.

In this article, participants were considered to have a SUD if they screened positive for hazardous, harmful or dependent alcohol on the AUDIT, or screened positive for hazardous, harmful or dependent drug use on the DUDIT, or clinically met the DSM-5 criteria for SUD by their treating doctor.

### Data analysis

Categorical variables were summarised by frequency and percentage tabulation, and continuous variables were described by the mean, standard deviation, median and interquartile range. These study variables were compared between the groups of patients with and without a SUD. The *χ*^2^ test was used to assess the relationships between categorical variables and SUD group. Fisher’s exact test was used for 2 × 2 tables or where the requirements for the *Χ*^2^ test could not be met. The relationship between continuous variables and the SUD group was assessed by the *t*-test. Where the data did not meet the assumptions of the test, a non-parametric alternative, the Wilcoxon rank sum test, was used. Data analysis was carried out using Statistical Analysis System (SAS) version 9.4 for Windows. The 5% significance level was used, meaning a *p*-value < 0.05 implies statistical significance.

### Ethical considerations

Ethical clearance was obtained from the Human Research Ethics Committee of the University of the Witwatersrand (HREC M151013).

## Results

A total of 177 patients were admitted over the 4-month period, from 01 February to 31 May 2016. Of these admissions, 10 had severe behavioural disturbance warranting immediate transfer to a specialist hospital for further involuntary care, four did not speak either English or Afrikaans, three lacked capacity to consent because of neurocognitive disorders and two were under the age of 18 years and thus were excluded and eight declined to participate. Following informed consent, the study population consisted of 150 participants.

### Demographic and clinical characteristics of the study population

Of the 150 participants, 62.7% (*n* = 94) were men, 76.7% (*n* = 115) were under the age of 40 years and 67.3% (*n* = 101) were black. Two-thirds (*n* = 99) of the participants were brought to hospital by a relative, parent or spouse; 16.0% (*n* = 24) by the police; and 7.3% (*n* = 11) by the ambulance (Table 1). For 40% (*n* = 60) of the participants this was their first psychiatric presentation. The remaining 60% (*n* = 90) were repeat presentations, with 44.0% (*n* = 66) of the study sample having two or more admissions in the last 2 year (Table 1).

**TABLE 1:** Socio-demographic characteristics of the sample.

Variable	Category	Number	%
Age	18–39 years	115	76.7
	40 years or more	35	23.3
Gender	Female	56	37.3
	Male	94	62.7
Population group	Black	101	67.3
	Mixed race	26	17.3
	White	20	13.3
	Indian	2	1.3
	Other	1	0.7
Relationship status	Single	87	58.0
	In a relationship	63	42.0
Highest level of education	None	1	0.7
	Primary	10	6.7
	High School	64	42.7
	Matric	51	34.0
	Tertiary	24	16.0
Employment status	Unemployed	81	54.0
	Employed	55	36.7
	Student or pensioner	14	9.3
Disability grant	No	127	84.7
	Yes	23	15.3
Presentation	Index to psychiatry	60	40.0
	Repeat	90	60.0
Number of admissions in the last 2 years	1	84	56.0
	2 or more	66	44.0

**FIGURE 1:**
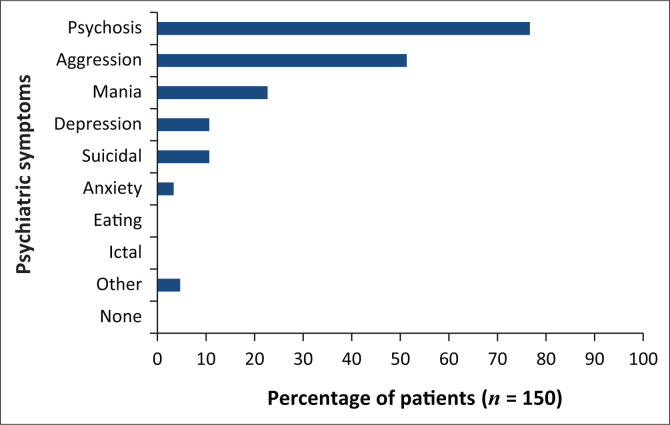
Psychiatric symptoms on admission.

Almost 80% (*n* = 115) of the participants presented with psychotic symptoms on admission and over 50% (*n* = 77) were aggressive (Figure 1). Rapid tranquillisation was necessary in 67% (*n* = 101) of the participants, of which approximately 15% (*n* = 22) required combinations of three or four tranquillising agents. According to the *Mental Health Care Act*, the admissions were predominately involuntary (58.7%, *n* = 88) or assisted (36.0%, *n* = 54), and the mean duration of hospital stay was 14 days (median 11). The most frequently occurring medical condition was HIV and AIDS, diagnosed in 20 participants, followed by hypertension (*n* = 6), epilepsy (*n* = 5), head injury (*n* = 4) and diabetes mellitus (*n* = 3).

### Clinical diagnosis of substance use disorders

**FIGURE 2:**
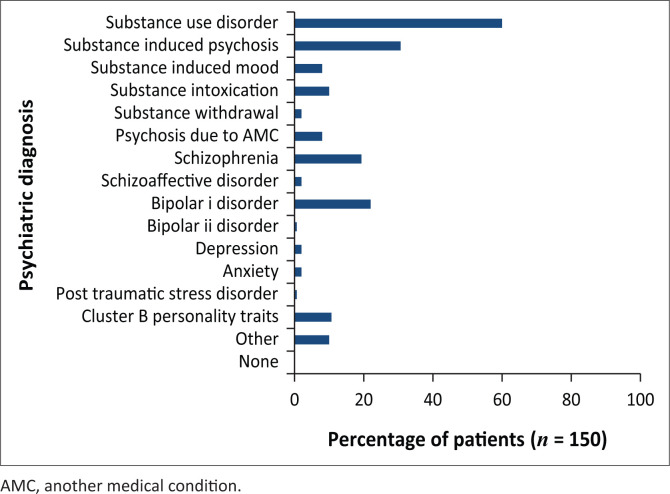
Clinical psychiatric diagnosis on discharge.

From the clinical notes by the treating doctor, it was observed that SUD was diagnosed in 60.0% (*n* = 90) of the participants. Substance-related conditions were the most frequent discharge diagnoses, with 30.7% (*n* = 46) of participants diagnosed with substance-induced psychotic disorder, 12.0% (*n* = 18) with intoxication or withdrawal, and 8.0% (*n* = 12) with substance-induced mood disorder (figure 2).

### Substance use disorders as determined by the AUDIT and DUDIT instruments

**FIGURE 3:**
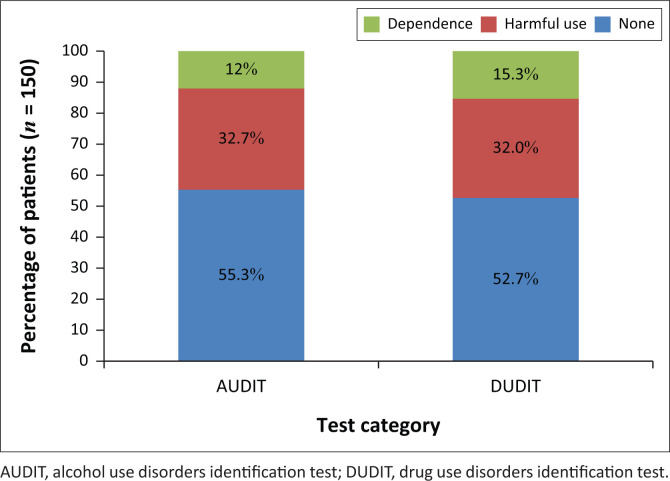
Percentage of sample in each alcohol use disorders identification test and drug use disorders identification test category.

Administration of the AUDIT showed that 44.6% (*n* = 67) of participants had an alcohol use disorder, and the DUDIT showed that 47.3% (*n* = 71) had a drug use disorder (Figure 3). A total of 25.2% (*n* = 38) of participants had both alcohol and drug use disorders. Overall, the AUDIT and DUDIT tools identified two-thirds of the participants (67.0%, *n* = 100) as having a current SUD (Table 2).

**TABLE 2:** Substance use comorbidity according to alcohol use disorders identification test and drug use disorders identification test categories.

AUDIT results	DUDIT results							
	No or low drug use		Hazardous or harmful use		Drug dependence		Total	
	*n*	%	*n*	%	*n*	%	*n*	%
No or low alcohol use	50	33.3	26	17.3	7	4.7	83	55.3
Hazardous or harmful use	21	14.0	17	11.3	11	7.3	49	32.7
Alcohol dependence	8	5.3	5	3.3	5	3.3	18	12

**Total**	**79**	**52.7**	**48**	**32.0**	**23**	**15.3**	**150**	**100**

AUDIT, Alcohol use disorders identification test; DUDIT, drug use disorders identification test.

### Comparison between AUDIT and DUDIT assessment with clinical diagnosis of substance use disorder

The findings of the two methods did not correspond in 23% (*n* = 36) of the participants: 15.3% (*n* = 23) of participants were identified with SUD by the questionnaire, but not clinically diagnosed with SUD. A total of 8.7% (*n* = 13) participants were clinically diagnosed with SUD, but did not have evidence of SUD on administration of the questionnaire (Table 3). Had the clinical diagnosis of SUD been augmented with the questionnaires, the overall yield would have been 75.3% (*n* = 113) SUD.

**TABLE 3:** Comparison of the alcohol use disorders identification test and drug use disorders identification test findings of substance use disorders with a clinical diagnosis of substance use disorders.

Substance use disorder (clinical diagnosis)	Substance use (AUDIT or DUDIT)					
	No SUD		SUD		Total	
	*n*	%	*n*	%	*n*	%
No SUD	37	24.7	23	15.3	60	40
SUD	13	8.7	77	51.3	90	60

**Total**	**50**	**33.3**	**100**	**66.7**	**150**	**100**

AUDIT, alcohol use disorders identification test; DUDIT, drug use disorders identification test; SUD, substance use disorders.

### Demographic and clinical characteristics associated with substance use disorders indicated on alcohol use disorders identification test and drug use disorders identification test

The demographic and clinical characteristics of those who screened positive for SUD by the AUDIT and DUDIT tools were compared with the group who did not (see Table 4).

**TABLE 4:** Comparison of characteristics between those without and with substance use disorders on alcohol use disorders identification test and drug use disorders identification test.

Variable	Category	No SUD		SUD		*p*
		*N* = 50	**%**	*N* = 100	**%**	
Age	18–39 years	30	60.0	85	85.0	0.0010*
	40 years and above	20	40.0	15	15.0	-
Gender	Female	26	52.0	30	30.0	0.0120*
	Male	24	48.0	70	70.0	-
Disability grant	No	37	74.0	90	90.0	0.0150*
	Yes	13	26.0	10	10.0	-
*Mental Health Care Act* status	Involuntary	25	50.0	63	63.0	0.0730
	Assisted	24	48.0	30	30.0	-
	Voluntary	1	2.0	7	7.0	-
Brought by	Family member	39	83.0	60	61.2	0.0250*
	Police	6	12.8	18	18.4	-
	Ambulance	1	2.1	10	10.2	-
	Self	1	2.1	10	10.2	-
	Other	2	4.0	3	3.0	-
Psychiatric diagnosis (working diagnosis in clinical records)	Substance use disorder	13	26.0	77	77.0	< 0.0001*
	Substance-induced psychotic disorder	5	10.0	41	41.0	< 0.0001*
	Substance-induced mood disorder	0	0.0	12	12.0	0.0088*
	Substance intoxication	1	2.0	14	14.0	0.0210*
	Substance withdrawal	0	0.0	3	3.0	0.5500
	Psychotic owing to medical condition	5	10.0	7	7.0	0.5400
	Schizophrenia	17	34.0	12	12.0	0.0020*
	Schizoaffective disorder	1	2.0	2	2.0	> 0.9900
	Bipolar 1 disorder	18	36.0	15	15.0	0.0059*
	Bipolar 2 disorder	0	0.0	1	1.0	> 0.9900
	Major depressive disorder	0	0.0	3	3.0	0.5500
	Anxiety	0	0.0	3	3.0	0.5500
	Post-traumatic stress	1	2.0	0	0.0	0.3300
	Personality disorder	4	8.0	12	12.0	0.5800
	Other	2	4.0	12	12.0	-
Medical comorbidity	HIV	8	16.0	12	12.0	0.4500
	Epilepsy	2	4.0	3	3.0	> 0.9900
	Head injury	1	2.0	3	3.0	> 0.9900
	Hypertension	3	6.0	3	3.0	0.4000
	Diabetes	1	2.0	2	2.0	> 0.9900
	None	36	72.0	80	80.0	0.3000
Presentation	Index	15	30.0	45	45.0	0.0810
	Repeat presentation	35	70.0	55	55.0	-
Number of admissions in the last 2 years	1	25	50.0	59	59.0	0.4800
	2 or more	25	50.0	41	41.0	-

SUD, substance use disorders.

* , Significant differences (*p* < 0.05).

There was a statistically significant association between SUD and younger age (*p* = 0.0010), male gender (*p* = 0.012) and being more likely to arrive at hospital with police or ambulance escort, or patients by themselves rather than by a family member (*p* = 0.025). Although unemployment was similar amongst those with SUD (56%) and those without SUD (50%), those with a SUD were significantly less likely to receive a disability grant (10% vs. 26%) (*p* = 0.015). There was no statistical difference in mental healthcare act status between the two groups (*p* = 0.073).

Those with a SUD on AUDIT or DUDIT were more likely to have a clinical diagnosis of SUD or a substance-related condition (*p* < 0.0001). Although those with SUD were less frequently diagnosed with schizophrenia or bipolar I disorder than those without SUD (*p* = 0.002 and *p* = 0.0059, respectively), 41.4% of participants with schizophrenia and 47.1% of with bipolar disorder admitted to have comorbid SUD.

A total of 72.7% participants (*n* = 72 SUD, *n* = 37 non-SUD) were discharged to home, 10.7% (*n* = 14 SUD, *n* = 2 non-SUD) were transferred to Sterkfontein Hospital (a restrictive psychiatric institution) for further involuntary care, 7.3% were transferred for further inpatient care at a less restrictive psychiatric hospital (*n* = 6 SUD, *n* = 5 non-SUD) and 8.0% (*n* = 7 SUD, *n* = 5 non-SUD) went to a long-stay medium-care facility. Two participants were referred to an inpatient substance rehabilitation centre upon discharge from hospital, which was arranged privately by the family members of the participants.

## Discussion

In this cross-sectional study of 150 acute psychiatric inpatients, we aimed to describe the prevalence of SUD and their clinical correlates using standardised screening instruments. A total of 67% of participants were identified as having SUD upon administration of the AUDIT and DUDIT questionnaires. Alcohol use was identified in 45% patients, other drug use in 47%, with evidence of comorbid use of alcohol and other drugs in 25% patients.

In South Africa, variable prevalence rates of SUD amongst psychiatric inpatients have been found.^10,14,29,30,31^ Our results are similar to the 62% prevalence of a current SUD found amongst acute psychiatric patients in the Western Cape,^29^ and to the 72% prevalence found amongst a sample of Gauteng state patients.^14^ Using the ASSIST questionnaire in a study at a regional psychiatric hospital in KwaZulu-Natal, South Africa,^22^ patients were reported to have a similar 54% recent alcohol use and 43% recent cannabis use.

This study revealed a higher prevalence of SUD than that found in a previous study conducted in the same unit.^31^ Analysing records of 520 patients admitted during 2007/2008 financial year, Janse van Rensburg^31^ found substance abuse documented in 40% of files and a substance-related condition diagnosed in 21% of cases. Their sample consisted of 49.8% men and 50.2% women, and schizophrenia was the most commonly diagnosed condition. Thus, 8 years later in the same unit, our sample showed a higher proportion of men and higher prevalence of SUD, which has superseded schizophrenia as the most common diagnosis.

However, the prevalence in our sample was less than the 81.2% found amongst patients admitted with first episode psychosis in the Eastern Cape.^30^ Substance use disorder has been shown to be more common with first episode psychosis than with subsequent episodes.^32^ Index psychiatric presentations were not frequent amongst those with SUD in our sample; however, if the 60 participants with an index psychiatric presentation were considered separately, then 75% (*n* = 45) had a SUD, bringing the prevalence rate closer to that found in the Eastern Cape study.^30^

When comparing to international prevalence rates of SUD, the prevalence of SUD in this study is higher than that reported in Iceland, which reported SUD in 58% of male and 32% of female psychiatric inpatients.^33^ Iceland is also experiencing an increase in SUD admissions in psychiatric units over the past 25 years, and this is more pronounced in substances other than alcohol.^34^ In the United States of America, two-thirds of inpatients with mental illness were reported to be either past substance abusers (26.1%) or current abusers (40.7%).^35^ Thus, although there are similar rates of lifetime SUD, this study has higher rates of ongoing SUD, as the AUDIT and DUDIT assess for current SUD.

In the overall sample, schizophrenia was diagnosed in 19.3% (*n* = 29) and bipolar disorder in 22.7% (*n* = 34) of the participants. Both were more frequently diagnosed amongst those without substance use; however, it is possible that some of the substance-induced psychotic (*n* = 46) and substance-induced mood (*n* = 12) disorders could be comorbid rather than induced. Nevertheless, the fact that 41.4% of those participants diagnosed with schizophrenia were found to have SUD is consistent with the finding of 41.7% prevalence rate of SUD amongst people with schizophrenia found by Hunt et al.^5^ Interestingly, Hunt et al.^5^ commented on the stability of cannabis and alcohol use prevalence amongst people with schizophrenia over time, being unchanging over a 27-year period. Amongst those with bipolar disorder, our 47.1% prevalence of SUD is higher than the 33% prevalence rate found by Hunt et al.^4^ in their systematic review of studies in clinical settings in high-income countries, confirming high rates of SUD amongst these psychiatric disorder patients. It is not known if this variation with international studies suggests differences in environmental, substance availability or illness factors.

In contrast, amongst general medical or surgical admissions, prevalence rates for alcohol (10%), cannabis (7%) and other substances (4.5%) have been reported^36^; these rates are much lower than the prevalence found amongst psychiatric patients. This varying prevalence highlights the importance of local surveys to explore the extent of substance use in individual psychiatric centres and implementing services according to the centre’s needs.

### Characteristics of those with substance use disorders

We found that those with SUD were more likely to be male and single person, which is consistent with the findings of local and international studies.^21,37^ Although 54% of the sample was unemployed, which is higher than the 2011 official 25% unemployment rate amongst the general population of Johannesburg,^38^ there was no difference between those with and without SUD. This may be related to a complicated relationship where mental illness increases the risk of unemployment,^39^ and unemployment may increase the risk of mental illness.^40^ The difference in disability grants may reflect differences in attitude to substance users by doctors, social workers and the officials approving grant applications or even the substance users’ inability to apply for disability grants.

Whilst the SUD group was more likely to be brought to hospital by police or ambulance, there was no increased rate of aggression, psychosis or mania, which are conditions that could result in a need for police or ambulance escort. Estrangement from family members as a result of the negative effects of SUD may be a possibility. Even though we did not confirm higher rates of psychosis and aggressive symptoms amongst those with SUD, 14 of the 16 participants referred for further involuntary care (usually related to aggressive, disruptive behaviour) screened positive for SUD. It is also not known if any of the 10 participants who were ineligible for study inclusion because of immediate transfer to Sterkfontein Hospital had used substances.

Despite high rates of substance use, only two participants were referred to inpatient rehabilitation centres upon discharge, which was arranged privately by their families This finding is consistent with the low referral rate described by the South African Community Epidemiology Network on Drug Use (SACENDU).^41^ In their data on help-seeking substance users from rehabilitation centres, Gauteng province statistics recorded 2734 admissions to 15 treatment centres in the first half of 2018, of which 59% were referred to the centres by themselves, families or friends; 14% by social workers; and 2% by a healthcare professional. Further exploration regarding accessibility of rehabilitation centres is required.

Psychiatric services generally do not offer substance rehabilitation. In South Africa, substance use management falls under the National Department of Social Development, which is obliged to implement inter-sectoral strategies for early detection and treatment under the *Prevention of and Treatment of Substance Use Act* No. 70 of 2008.^42^ In collaboration with the Department of Social Development and other government departments, the Department of Health is responsible for meeting the medical and psychiatric needs of substance users. Notably, the National Drug Master Plan,^43^ within the Department of Social Development, which should provide a blueprint for inter-sectoral engagement, expired at the end of 2017, and a new plan is in the process of development. Therefore, collaborative networks and referral pathways between the two departments are awaiting revision.

Clients with both psychiatric and SUD have reported greater satisfaction with an integrated dual diagnosis treatment approach.^44^ In Gauteng province, one government inpatient dual diagnosis unit is available at the remote, stand-alone Sterkfontein Psychiatric Hospital; however, this is not easily accessible, not all are candidates for such intensive programmes and no referrals were made to it from Helen Joseph Hospital during the study period. Outpatient government dual diagnosis services are available only at one central hospital, Chris Hani Baragwanath Hospital.

### Consistency between questionnaire and clinical diagnosis of substance use disorders

In 76% (*n* = 114) of the participants, the questionnaire and clinical diagnosis were consistent; 24.7% (*n* = 37) of the participants were non-SUD and 51.3% (*n* = 77) were SUD. However, 15.3% (*n* = 23) of patients screened positive for SUD upon administration of the questionnaire but were not identified clinically, and 8.7% (*n* = 13) of patients were not screened positive by the questionnaire but identified so on clinical assessment. Augmentation of clinical screening with a questionnaire would have improved the detection of SUD, a finding that is consistent with the recommendations made by Morejele et al. and WHO.^7,27,28^

## Limitations

Generalisability may be limited as this study was conducted at only one acute centre with a small sample size. A larger sample size could also have revealed more significant differences in clinical correlates. The screening tools used did not assess for which drugs were used. This is an important information as the wide range of available substances cause varying risks of addiction, severity of tolerance and different symptoms. The tools have been validated in English; however, the investigator’s translating the tool into Afrikaans is not a standardised practice. Language considerations should be made according to the centre’s needs.

## Conclusion and recommendations

Notwithstanding the limitations, our study adds to the growing body of evidence of a high prevalence of SUD amongst patients admitted to psychiatric units in South Africa, particularly young men. Two-thirds of the participants were identified with SUD. The AUDIT and DUDIT questionnaires are cost-effective and quick to administer. Consideration should be given to their routine use to enhance the clinical identification in populations with high numbers of substance users.

Despite the high prevalence of SUD, only two participants went to an inpatient rehabilitation facility following discharge. The severity of the SUD was not consistently noted by clinicians, which could inform whether inpatient or outpatient SUD rehabilitation services are required. Thus, we encourage the specification of disorders to improve clinical services. Further research should explore the referrals and follow-up to outpatient services. Although not all substance users require inpatient rehabilitation, the low referral rate may be partly because of psychiatric services and substance rehabilitation facilities being managed by different governmental departments. Thus, improved collaboration of services is recommended.
